# Correction to: Phage infection fronts trigger early sporulation and viral entrapment in bacterial populations

**DOI:** 10.1093/ismejo/wrag194

**Published:** 2026-08-20

This is a correction to: Andreea Măgălie, Anastasios Marantos, Joy M O’Brien, Daniel A Schwartz, Jacopo Marchi, Jay T Lennon, Joshua S Weitz, Phage infection fronts trigger early sporulation and viral entrapment in bacterial populations, *The ISME Journal*, Volume 20, Issue 1, January 2026, wrag023, https://doi.org/10.1093/ismejo/wrag023

In the originally published version of this manuscript, the caption for Figure 6 contained errors in the description of the results.

A sentence has been corrected to read:

“The results shown in the middle column panels and the solid line in the right column panels correspond to *t* = 5 h after phage addition, which lies within the time window of *t* ≈ 3–7 h during which a transient peak in sporulation around the plaque emerges in our simulations”.

Instead of:

“The results shown in the middle column panels and the solid line in the right column panels correspond to *t* = h after phage addition, which lies within the time window of approximately*t* ≈ 3–7 h during which a transient peak in sporulation around the plaque emerges in our simulations”.

